# 2-(1*H*-Indol-3-yl)-4,4,5,5-tetra­methyl­imidazolidine-1-oxyl 3-oxide

**DOI:** 10.1107/S1600536810002175

**Published:** 2010-01-23

**Authors:** Hai-Bo Wang, Lin-Lin Jing, Ru Jiang, Peng Liu, Xiao-Li Sun

**Affiliations:** a Department of Chemistry, School of Pharmacy, Fourth Military Medical University, Changle West Road 17, 710032 Xi-An, People’s Republic of China

## Abstract

In the title compound, C_15_H_18_N_3_O_2_, the plane of the indole ring system is twisted with respect to the plane of the nitronyl nitroxide moiety, exhibiting a dihedral angle of 21.61 (6)°. The crystal packing is stabilized by N—H⋯O hydrogen bonds and weak C—H⋯O inter­actions.

## Related literature

For the preparation of nitronyl nitroxides, see: Ullman *et al.* (1974 ▶). For their biological activity, see: Soule *et al.* (2007 ▶) and their coordination properties, see: Masuda *et al.* (2009 ▶). For related structures, see: Iqbal *et al.* (2009 ▶); Qin *et al.* (2009 ▶); Tanaka *et al.* (2007 ▶).
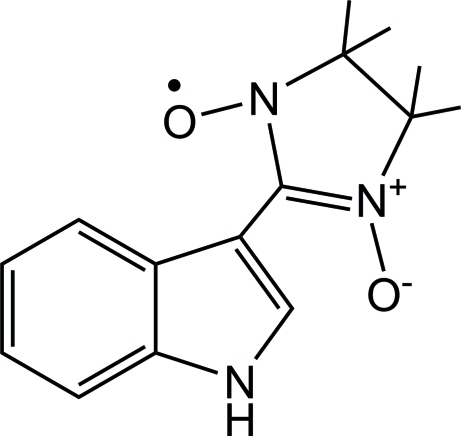


## Experimental

### 

#### Crystal data

C_15_H_18_N_3_O_2_
*M* *_r_* = 272.32
Orthorhombic, 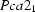

*a* = 15.0810 (15) Å
*b* = 8.7700 (8) Å
*c* = 10.6108 (10) Å
*V* = 1403.4 (2) Å^3^
*Z* = 4
Mo *K*α radiation
μ = 0.09 mm^−1^
*T* = 296 K
0.37 × 0.29 × 0.18 mm

#### Data collection

Bruker APEXII CCD area-detector diffractometer
Absorption correction: multi-scan (*SADABS*; Bruker, 2007 ▶) *T*_min_ = 0.968, *T*_max_ = 0.984
6651 measured reflections
1323 independent reflections
1208 reflections with *I* > 2σ(*I*)
*R*_int_ = 0.023

#### Refinement

*R*[*F* ^2^ > 2σ(*F* ^2^)] = 0.025
*wR*(*F* ^2^) = 0.067
*S* = 1.06
1323 reflections
186 parameters
1 restraint
H-atom parameters constrained
Δρ_max_ = 0.13 e Å^−3^
Δρ_min_ = −0.09 e Å^−3^

### 

Data collection: *APEX2* (Bruker, 2007 ▶); cell refinement: *SAINT* (Bruker, 2007 ▶); data reduction: *SAINT*; program(s) used to solve structure: *SHELXS97* (Sheldrick, 2008 ▶); program(s) used to refine structure: *SHELXL97* (Sheldrick, 2008 ▶); molecular graphics: *ORTEP-3* (Farrugia, 1997 ▶) and *DIAMOND* (Brandenburg, 1998 ▶); software used to prepare material for publication: *SHELXL97* and *PLATON* (Spek, 2009 ▶).

## Supplementary Material

Crystal structure: contains datablocks I, global. DOI: 10.1107/S1600536810002175/fl2283sup1.cif

Structure factors: contains datablocks I. DOI: 10.1107/S1600536810002175/fl2283Isup2.hkl

Additional supplementary materials:  crystallographic information; 3D view; checkCIF report

## Figures and Tables

**Table 1:** Hydrogen-bond geometry (Å, °)

*D*—H⋯*A*	*D*—H	H⋯*A*	*D*⋯*A*	*D*—H⋯*A*
N1—H1⋯O2^i^	0.86	2.07	2.8506 (18)	150
C12—H12*C*⋯O2^ii^	0.96	2.51	3.434 (2)	161
C14—H14*C*⋯O1^iii^	0.96	2.56	3.495 (2)	164

Symmetry codes: (i) 
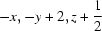
; (ii) 
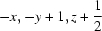
; (iii) 
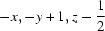
.

**Fig. 1.**
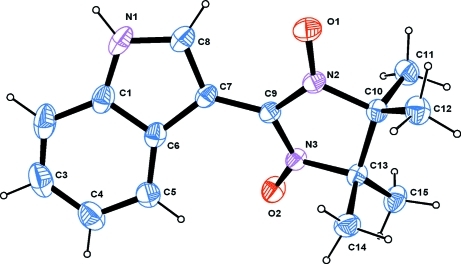
Molecular structure of the title compound (I), showing the atom labeling scheme. Displacement ellipsoids are drawn at the 30% probability level.

## supplementary crystallographic information

### Comment

Nitronyl nitroxides, stable organic radicals, that were originally synthesized more than 30 years ago (Ullman *et al.*1974), have recently received considerable attention (Iqbal *et al.* 2009; Qin *et al.* 2009; Tanaka *et al. *2007) because of their biological properities as anticancer, antiradiation and antioxidation**(Soule *et al.*, 2007). The title compound itself can be used to form coordination compounds with many metal cations, such as Mn^2+^, Cu^2+^ and Ni^2+^ leading to some interesting magentic materials (Masuda, *et al.*, 2009). The molecular structure of the title compound is shown in Fig1. The indole moiety and the nitronyl nitroxide ring are twisted with respect to each other making a dihedral angle of 21.6 (6)°. One of the  oxygen atoms (O2) of the nitronyl nitroxide moietie acts as an acceptor in a hydrogen bond from the N—H group of an adjacent molecule and both oxygens (O1 and O2) are acceptors in weak C-H···O intermolecular interactions that help stabilize the crystal packing (Table 1).

### Experimental

2,3-Dimethyl-2,3-bis(hydroxylamino) butane (1.48 g, 10.0 mmol) and 1*H*-indoline-3-carbaldehyde (1.47 g, 10.0 mmol) were dissolved in methanol. The reaction was stirred for 15 h at reflux temperature, then cooled to room temperature and filtered. The resulting white powder was washed by methanol and suspended in a mixed solution of dichloromethane (30.0 ml) and water (30.0 ml). Then the reaction mixture was added to an aqueous solution of NaIO_4_ and stirred for 15 min in an ice bath to give a blue solution. The aqueous phase was extracted with CH_2_Cl_2_ and the organic layer was combined and dried over MgSO_4_. Then the solvent was removed to give a dark blue residue which was purified by flash column chromatography with the elution of *n*-hexane/ ethyl acetate (1:3) to yield the title compound (I) as a dark blue powder. Single crystals of (I) were obtained from a mixed solution of *n*-heptane and dichloromethane (the ratio of volume is 1 to 1).

### Refinement

In the structure, all the H atoms were discernible in the difference Fourier maps. However, they were constrained by riding model approximation. *C*—H_methyl_=0.96 Å; *C*—H_aryl_=0.93 Å; *U*_iso_H_methyl_ and *U*_iso_H_aryl_ are 1.5 U _eq_(C) and 1.2 U _eq _(C), respectively. Since it was not possible to obtain information on the handedness of the molecule from the experimental data the Friedel euivalents were merged before the final cycles of refinement.

### Crystal data

**Table:**

C_15_H_18_N_3_O_2_	*F*(000) = 580
*M**_r_* = 272.32	*D*_x_ = 1.289 Mg m^−^^3^
Orthorhombic, *P**c**a*2_1_	Mo *K*α radiation, λ = 0.71073 Å
Hall symbol: P 2c -2ac	Cell parameters from 2598 reflections
*a* = 15.0810 (15) Å	θ = 2.7–24.4°
*b* = 8.7700 (8) Å	µ = 0.09 mm^−^^1^
*c* = 10.6108 (10) Å	*T* = 296 K
*V* = 1403.4 (2) Å^3^	Block, blue
*Z* = 4	0.37 × 0.29 × 0.18 mm

### Data collection

**Table:**

Bruker APEXII CCD area-detector diffractometer	1323 independent reflections
Radiation source: fine-focus sealed tube	1208 reflections with *I* > 2σ(*I*)
graphite	*R*_int_ = 0.023
phi and ω scans	θ_max_ = 25.1°, θ_min_ = 2.3°
Absorption correction: multi-scan (*SADABS*; Bruker, 2007)	*h* = −14→17
*T*_min_ = 0.968, *T*_max_ = 0.984	*k* = −8→10
6651 measured reflections	*l* = −12→12

### Refinement

**Table:**

Refinement on *F*^2^	Secondary atom site location: difference Fourier map
Least-squares matrix: full	Hydrogen site location: inferred from neighbouring sites
*R*[*F*^2^ > 2σ(*F*^2^)] = 0.025	H-atom parameters constrained
*wR*(*F*^2^) = 0.067	*w* = 1/[σ^2^(*F*_o_^2^) + (0.0394*P*)^2^ + 0.1053*P*] where *P* = (*F*_o_^2^ + 2*F*_c_^2^)/3
*S* = 1.06	(Δ/σ)_max_ < 0.001
1323 reflections	Δρ_max_ = 0.13 e Å^−^^3^
186 parameters	Δρ_min_ = −0.09 e Å^−^^3^
1 restraint	Extinction correction: *SHELXL97* (Sheldrick, 2008), Fc^*^=kFc[1+0.001xFc^2^λ^3^/sin(2θ)]^-1/4^
Primary atom site location: structure-invariant direct methods	Extinction coefficient: 0.0146 (19)

### Special details

**Table:**

Geometry. All e.s.d.'s (except the e.s.d. in the dihedral angle between two l.s. planes) are estimated using the full covariance matrix. The cell e.s.d.'s are taken into account individually in the estimation of e.s.d.'s in distances, angles and torsion angles; correlations between e.s.d.'s in cell parameters are only used when they are defined by crystal symmetry. An approximate (isotropic) treatment of cell e.s.d.'s is used for estimating e.s.d.'s involving l.s. planes.
Refinement. Refinement of *F*^2^ against ALL reflections. The weighted *R*-factor *wR* and goodness of fit *S* are based on *F*^2^, conventional *R*-factors *R* are based on *F*, with *F* set to zero for negative *F*^2^. The threshold expression of *F*^2^ > σ(*F*^2^) is used only for calculating *R*-factors(gt) *etc*. and is not relevant to the choice of reflections for refinement. *R*-factors based on *F*^2^ are statistically about twice as large as those based on *F*, and *R*- factors based on ALL data will be even larger.we could not determine the absolute configuration,because there is no atom heavier than Si in the molecular

### Fractional atomic coordinates and isotropic or equivalent isotropic displacement parameters (Å^2^)

**Table:**

	*x*	*y*	*z*	*U*_iso_*/*U*_eq_	
N1	−0.01319 (14)	1.0261 (2)	0.9413 (2)	0.0605 (5)	
H1	−0.0087	1.0928	1.0004	0.073*	
N2	0.11909 (10)	0.60749 (18)	0.83997 (15)	0.0393 (4)	
N3	0.05128 (10)	0.62465 (17)	0.65920 (15)	0.0381 (4)	
O1	0.14925 (11)	0.64081 (18)	0.94892 (14)	0.0579 (4)	
O2	0.01465 (11)	0.68277 (17)	0.56075 (14)	0.0545 (4)	
C1	−0.08282 (15)	1.0156 (2)	0.8579 (2)	0.0534 (6)	
C2	−0.16017 (18)	1.1028 (3)	0.8506 (3)	0.0731 (8)	
H2	−0.1707	1.1825	0.9064	0.088*	
C3	−0.21965 (16)	1.0666 (3)	0.7587 (4)	0.0795 (9)	
H3	−0.2715	1.1234	0.7516	0.095*	
C4	−0.20480 (15)	0.9465 (3)	0.6750 (3)	0.0692 (7)	
H4	−0.2463	0.9255	0.6126	0.083*	
C5	−0.12892 (14)	0.8583 (2)	0.6839 (2)	0.0529 (6)	
H5	−0.1201	0.7769	0.6292	0.063*	
C6	−0.06560 (13)	0.8930 (2)	0.7760 (2)	0.0442 (5)	
C7	0.01895 (13)	0.8304 (2)	0.81520 (18)	0.0407 (5)	
C8	0.04679 (15)	0.9165 (2)	0.9167 (2)	0.0515 (5)	
H8	0.0991	0.9010	0.9614	0.062*	
C9	0.06218 (12)	0.6932 (2)	0.77161 (18)	0.0363 (4)	
C10	0.15347 (12)	0.4734 (2)	0.76668 (19)	0.0385 (4)	
C11	0.24518 (16)	0.5183 (2)	0.7176 (2)	0.0523 (5)	
H11A	0.2395	0.6018	0.6597	0.079*	
H11B	0.2715	0.4328	0.6752	0.079*	
H11C	0.2821	0.5483	0.7870	0.079*	
C12	0.15962 (15)	0.3356 (2)	0.8531 (2)	0.0516 (5)	
H12A	0.2043	0.3532	0.9158	0.077*	
H12B	0.1749	0.2471	0.8045	0.077*	
H12C	0.1035	0.3195	0.8936	0.077*	
C13	0.08199 (13)	0.4624 (2)	0.66190 (19)	0.0381 (4)	
C14	0.00154 (14)	0.3668 (2)	0.6989 (2)	0.0516 (5)	
H14A	−0.0183	0.3965	0.7813	0.077*	
H14B	0.0176	0.2609	0.6997	0.077*	
H14C	−0.0453	0.3829	0.6391	0.077*	
C15	0.11578 (16)	0.4160 (3)	0.5331 (2)	0.0556 (6)	
H15A	0.0674	0.4150	0.4743	0.083*	
H15B	0.1415	0.3161	0.5379	0.083*	
H15C	0.1598	0.4876	0.5054	0.083*	

### Atomic displacement parameters (Å^2^)

**Table:**

	*U*^11^	*U*^22^	*U*^33^	*U*^12^	*U*^13^	*U*^23^
N1	0.0810 (13)	0.0386 (10)	0.0620 (12)	0.0000 (9)	0.0137 (11)	−0.0174 (10)
N2	0.0440 (9)	0.0423 (8)	0.0315 (8)	−0.0009 (7)	0.0003 (7)	−0.0031 (7)
N3	0.0481 (9)	0.0330 (8)	0.0333 (8)	0.0030 (7)	−0.0032 (7)	0.0023 (7)
O1	0.0670 (9)	0.0679 (10)	0.0389 (8)	0.0047 (7)	−0.0128 (7)	−0.0112 (8)
O2	0.0774 (10)	0.0452 (8)	0.0407 (8)	0.0126 (7)	−0.0113 (8)	0.0051 (6)
C1	0.0613 (13)	0.0344 (11)	0.0644 (15)	−0.0023 (9)	0.0182 (12)	−0.0009 (10)
C2	0.0771 (18)	0.0385 (12)	0.104 (2)	0.0098 (11)	0.0325 (17)	0.0013 (14)
C3	0.0534 (14)	0.0547 (14)	0.130 (3)	0.0105 (11)	0.0192 (18)	0.0178 (18)
C4	0.0492 (13)	0.0600 (14)	0.098 (2)	−0.0007 (11)	0.0023 (14)	0.0130 (15)
C5	0.0485 (11)	0.0439 (11)	0.0663 (15)	−0.0032 (9)	0.0053 (11)	0.0049 (11)
C6	0.0487 (10)	0.0304 (10)	0.0535 (12)	−0.0032 (8)	0.0117 (10)	0.0034 (9)
C7	0.0485 (11)	0.0329 (10)	0.0406 (11)	−0.0040 (8)	0.0070 (8)	−0.0021 (8)
C8	0.0614 (13)	0.0411 (11)	0.0520 (13)	−0.0036 (10)	0.0062 (11)	−0.0070 (10)
C9	0.0405 (9)	0.0333 (9)	0.0351 (10)	−0.0032 (8)	0.0014 (8)	0.0005 (9)
C10	0.0418 (10)	0.0375 (10)	0.0363 (9)	0.0025 (7)	0.0029 (8)	0.0009 (9)
C11	0.0440 (10)	0.0571 (12)	0.0560 (12)	−0.0003 (10)	0.0081 (10)	−0.0009 (12)
C12	0.0584 (13)	0.0488 (12)	0.0474 (12)	0.0065 (9)	−0.0017 (10)	0.0091 (11)
C13	0.0464 (10)	0.0323 (9)	0.0357 (9)	0.0031 (8)	0.0015 (8)	−0.0008 (8)
C14	0.0540 (11)	0.0415 (11)	0.0592 (13)	−0.0070 (9)	−0.0065 (11)	−0.0003 (10)
C15	0.0734 (15)	0.0521 (12)	0.0412 (12)	0.0127 (11)	0.0007 (11)	−0.0085 (10)

### Geometric parameters (Å, °)

**Table:**

N1—C8	1.346 (3)	C7—C8	1.381 (3)
N1—C1	1.377 (3)	C7—C9	1.444 (3)
N1—H1	0.8600	C8—H8	0.9300
N2—O1	1.276 (2)	C10—C12	1.519 (3)
N2—C9	1.352 (2)	C10—C11	1.529 (3)
N2—C10	1.502 (2)	C10—C13	1.552 (3)
N3—O2	1.287 (2)	C11—H11A	0.9600
N3—C9	1.346 (2)	C11—H11B	0.9600
N3—C13	1.496 (2)	C11—H11C	0.9600
C1—C2	1.397 (3)	C12—H12A	0.9600
C1—C6	1.407 (3)	C12—H12B	0.9600
C2—C3	1.362 (5)	C12—H12C	0.9600
C2—H2	0.9300	C13—C15	1.515 (3)
C3—C4	1.396 (4)	C13—C14	1.526 (3)
C3—H3	0.9300	C14—H14A	0.9600
C4—C5	1.384 (3)	C14—H14B	0.9600
C4—H4	0.9300	C14—H14C	0.9600
C5—C6	1.400 (3)	C15—H15A	0.9600
C5—H5	0.9300	C15—H15B	0.9600
C6—C7	1.449 (3)	C15—H15C	0.9600
			
C8—N1—C1	109.91 (19)	N2—C10—C12	109.33 (16)
C8—N1—H1	125.0	N2—C10—C11	106.68 (15)
C1—N1—H1	125.0	C12—C10—C11	110.80 (17)
O1—N2—C9	125.79 (16)	N2—C10—C13	100.34 (14)
O1—N2—C10	121.68 (16)	C12—C10—C13	115.19 (16)
C9—N2—C10	112.14 (16)	C11—C10—C13	113.58 (17)
O2—N3—C9	126.49 (15)	C10—C11—H11A	109.5
O2—N3—C13	121.66 (15)	C10—C11—H11B	109.5
C9—N3—C13	111.73 (15)	H11A—C11—H11B	109.5
N1—C1—C2	129.5 (2)	C10—C11—H11C	109.5
N1—C1—C6	107.89 (19)	H11A—C11—H11C	109.5
C2—C1—C6	122.6 (3)	H11B—C11—H11C	109.5
C3—C2—C1	117.5 (3)	C10—C12—H12A	109.5
C3—C2—H2	121.2	C10—C12—H12B	109.5
C1—C2—H2	121.2	H12A—C12—H12B	109.5
C2—C3—C4	121.7 (2)	C10—C12—H12C	109.5
C2—C3—H3	119.2	H12A—C12—H12C	109.5
C4—C3—H3	119.2	H12B—C12—H12C	109.5
C5—C4—C3	120.7 (3)	N3—C13—C15	110.02 (16)
C5—C4—H4	119.6	N3—C13—C14	106.34 (15)
C3—C4—H4	119.6	C15—C13—C14	110.62 (19)
C4—C5—C6	119.3 (2)	N3—C13—C10	99.78 (14)
C4—C5—H5	120.3	C15—C13—C10	115.44 (17)
C6—C5—H5	120.3	C14—C13—C10	113.69 (17)
C5—C6—C1	118.12 (19)	C13—C14—H14A	109.5
C5—C6—C7	135.90 (19)	C13—C14—H14B	109.5
C1—C6—C7	105.97 (19)	H14A—C14—H14B	109.5
C8—C7—C9	124.61 (19)	C13—C14—H14C	109.5
C8—C7—C6	106.51 (17)	H14A—C14—H14C	109.5
C9—C7—C6	128.38 (18)	H14B—C14—H14C	109.5
N1—C8—C7	109.7 (2)	C13—C15—H15A	109.5
N1—C8—H8	125.1	C13—C15—H15B	109.5
C7—C8—H8	125.1	H15A—C15—H15B	109.5
N3—C9—N2	107.72 (15)	C13—C15—H15C	109.5
N3—C9—C7	126.94 (17)	H15A—C15—H15C	109.5
N2—C9—C7	125.30 (17)	H15B—C15—H15C	109.5
			
C8—N1—C1—C2	178.2 (2)	C10—N2—C9—C7	−179.20 (17)
C8—N1—C1—C6	−0.2 (2)	C8—C7—C9—N3	−163.74 (19)
N1—C1—C2—C3	−179.1 (2)	C6—C7—C9—N3	25.5 (3)
C6—C1—C2—C3	−0.9 (4)	C8—C7—C9—N2	18.9 (3)
C1—C2—C3—C4	0.4 (4)	C6—C7—C9—N2	−151.90 (19)
C2—C3—C4—C5	0.8 (4)	O1—N2—C10—C12	45.8 (2)
C3—C4—C5—C6	−1.6 (4)	C9—N2—C10—C12	−141.06 (17)
C4—C5—C6—C1	1.1 (3)	O1—N2—C10—C11	−74.1 (2)
C4—C5—C6—C7	179.4 (2)	C9—N2—C10—C11	99.07 (18)
N1—C1—C6—C5	178.64 (19)	O1—N2—C10—C13	167.26 (16)
C2—C1—C6—C5	0.1 (3)	C9—N2—C10—C13	−19.56 (19)
N1—C1—C6—C7	−0.1 (2)	O2—N3—C13—C15	34.2 (3)
C2—C1—C6—C7	−178.6 (2)	C9—N3—C13—C15	−149.50 (18)
C5—C6—C7—C8	−178.0 (2)	O2—N3—C13—C14	−85.6 (2)
C1—C6—C7—C8	0.4 (2)	C9—N3—C13—C14	90.7 (2)
C5—C6—C7—C9	−6.0 (4)	O2—N3—C13—C10	155.98 (16)
C1—C6—C7—C9	172.47 (19)	C9—N3—C13—C10	−27.72 (19)
C1—N1—C8—C7	0.5 (2)	N2—C10—C13—N3	25.95 (17)
C9—C7—C8—N1	−172.98 (19)	C12—C10—C13—N3	143.19 (16)
C6—C7—C8—N1	−0.5 (2)	C11—C10—C13—N3	−87.50 (18)
O2—N3—C9—N2	−167.34 (17)	N2—C10—C13—C15	143.77 (17)
C13—N3—C9—N2	16.6 (2)	C12—C10—C13—C15	−99.0 (2)
O2—N3—C9—C7	14.9 (3)	C11—C10—C13—C15	30.3 (2)
C13—N3—C9—C7	−161.20 (17)	N2—C10—C13—C14	−86.84 (18)
O1—N2—C9—N3	175.82 (17)	C12—C10—C13—C14	30.4 (2)
C10—N2—C9—N3	3.0 (2)	C11—C10—C13—C14	159.70 (17)
O1—N2—C9—C7	−6.4 (3)		

### Hydrogen-bond geometry (Å, °)

**Table:**

*D*—H···*A*	*D*—H	H···*A*	*D*···*A*	*D*—H···*A*
N1—H1···O2^i^	0.86	2.07	2.8506 (18)	150
C12—H12C···O2^ii^	0.96	2.51	3.434 (2)	161
C14—H14C···O1^iii^	0.96	2.56	3.495 (2)	164

Symmetry codes: (i) −*x*, −*y*+2, *z*+1/2; (ii) −*x*, −*y*+1, *z*+1/2; (iii) −*x*, −*y*+1, *z*−1/2.
